# Supplementary material for: A Preclinical Evaluation towards the Clinical Application of Oxygen Consumption Measurement by CERMs by a Mouse Chimera Model
Source: Int J Mol Sci. 2019 Nov 12;20(22):5650. doi: 10.3390/ijms20225650 (PMC6888687; doi:10.3390/ijms20225650)
Supplement: Supplementary file 1 [file ijms-20-05650-s001.zip › Kuno et al., IJMS suppl/Kuno et al., sapplemental Table 1.docx]

Supplemental Table 1.

Details of 8 replicated embryo transfers based on OCR

|  |  | transfer side | # of embryos  transferred | Mean OCR  (fmol/s) | Mean diameter  (μm) | # of  implantations | # of fetuses | average fetus weight | average placental weight |
| --- | --- | --- | --- | --- | --- | --- | --- | --- | --- |
| #1 | High OCR | L | 10 | 16.33 | 113.8 | 4 | 4 | 80.9 | 81.8 |
|  | Low OCR | R | 10 | 9.37 | 125.7 | 0 | 0 | NA | NA |
| #2 | High OCR | L | 10 | 11.98 | 104.9 | 2 | 0 | NA | NA |
|  | Low OCR | R | 10 | 6.70 | 104.5 | 7 | 2 | 94.3 | 97.4 |
| #3 | High OCR | L | 10 | 14.60 | 110.4 | 6 | 1 | 106.9 | 79.2 |
|  | Low OCR | R | 10 | 7.80 | 96.8 | 8 | 6 | 103.4 | 93.6 |
| #4 | High OCR | L | 10 | 13.38 | 120.8 | 7 | 2 | 108.9 | 98.3 |
|  | Low OCR | R | 10 | 7.69 | 112.5 | 7 | 4 | 80.0 | 84.6 |
| #5 | High OCR | R | 10 | 14.24 | 120.8 | 6 | 2 | 98.2 | 76.2 |
|  | Low OCR | L | 10 | 6.82 | 120.9 | 8 | 7 | 97.5 | 97.3 |
| #6 | High OCR | R | 10 | 18.34 | 107.9 | 6 | 3 | 48.6 | 76.2 |
|  | Low OCR | L | 10 | 7.74 | 103.7 | 3 | 0 | NA | NA |
| #7 | High OCR | L | 10 | 8.89 | 101.5 | 7 | 2 | 71.4 | 90.7 |
|  | Low OCR | R | 10 | 5.10 | 107.7 | 9 | 4 | 66.8 | 107.1 |

*NA: Not applicable

Legend

Table shows details of 8 replicated embryo transfers based on OCR in which supplement Table 1 in the main text. An experiment #1 was excluded because technical failure occurred in one of the transfers and no implantation was observed in one side of the bicornuate uterus.
