# Supplementary material for: A Preclinical Evaluation towards the Clinical Application of Oxygen Consumption Measurement by CERMs by a Mouse Chimera Model
Source: Int J Mol Sci. 2019 Nov 12;20(22):5650. doi: 10.3390/ijms20225650 (PMC6888687; doi:10.3390/ijms20225650)
Supplement: Supplementary file 1 [file ijms-20-05650-s001.zip › Kuno et al., IJMS suppl/Kuno et al., Sapplemental Table 2.docx]

Sapplmental Table 2.

Details of 4 replicated embryo transfers based on morphology (size)

|  |  | transfer side | # of embryos  transferred | Mean OCR  (fmol/s) | Mean diameter  (μm) | # of implantations | # of fetuses | average fetus weight (g) | average placental weight (g) |
| --- | --- | --- | --- | --- | --- | --- | --- | --- | --- |
| #1 | Large | L | 10 | 8.39 | 151.2 | 10 | 7 | 74.1 | 66.0 |
|  | Small | R | 10 | 9.33 | 121.8 | 7 | 3 | 69.2 | 68.7 |
| #2 | Large | R | 10 | 8.74 | 129.7 | 2 | 1 | 124.1 | 114.7 |
|  | Small | L | 10 | 10.64 | 102.1 | 4 | 1 | 131.3 | 80.3 |
| #3 | Large | L | 10 | 10.72 | 124.6 | 0 | 0 | NA | NA |
|  | Small | R | 10 | 10.76 | 101.3 | 4 | 0 | NA | NA |
| #4 | Large | R | 10 | 12.7 | 123.5 | 8 | 5 | 51.6 | 72.8 |
|  | Small | L | 10 | 12.45 | 91.5 | 6 | 4 | 59.5 | 67.5 |

*NA: Not applicable

Legend

Table shows details of 4 replicated embryo transfers based on morphology (size) in which supplement Table 2 in the main text. An experiment #3 was excluded because technical failure occurred in one of the transfers and no implantation was observed in one side of the bicornuate uterus.
